# Preemptive regional nerve blocks for sternotomy in pediatric cardiac surgery: a Bayesian network meta-analysis

**DOI:** 10.1016/j.bjane.2025.844652

**Published:** 2025-06-20

**Authors:** Bruno F.M. Wegner, Gustavo R.M. Wegner, Jaime A. Arias, Tatiana S. Nascimento

**Affiliations:** a Universidade Federal do Rio Grande do Sul, Porto Alegre, RS, Brazil; b Universidade Federal da Fronteira Sul, Passo Fundo, RS, Brazil; c Hospital Ana Nery, Salvador, BA, Brazil; d University of Iowa, Iowa City, USA

**Keywords:** Cardiovascular anesthesia, Network meta-analysis, Pediatric anesthesia, Perioperative care, Regional anesthesia

## Abstract

**Background:**

Effective pain management and expedited recovery are critical in pediatric cardiac surgery. While regional anesthesia techniques provide targeted pain control and may reduce opioid use and related complications, comparative evidence among regional nerve blocks in this population is limited. This study aimed to conduct a systematic review and network meta-analysis to support clinical decision-making for optimal analgesia.

**Methods:**

We conducted a Bayesian Network Meta-Analysis (NMA) including Randomized Controlled Trials (RCTs) of pediatric patients (0–12 years) undergoing cardiac surgery by sternotomy and receiving preemptive regional nerve blocks. Primary outcomes included pain scores, opioid consumption and extubation time. Both direct and indirect evidence were synthesized to rank interventions probabilistically. This study was registered on PROSPERO (CRD42024585785) and followed PRISMA Extension Statement for Reporting of Systematic Reviews Incorporating Network Meta-analyses of Health Care Interventions.

**Results:**

The NMA incorporated 12 RCTs, comprising 969 participants, and evaluated seven regional nerve blocks. Among the techniques studied, transversus Thoracis Muscle Plane Block (TTPB) consistently ranked among the most effective for pain relief and recovery. Other blocks, including thoracic retrolaminar block and thoracic paravertebral block, also demonstrated notable performances. Adverse events were infrequent but inconsistently reported, preventing an adequate analysis.

**Conclusion:**

This NMA identified TTPB as a consistently top-performing technique across outcomes. These findings provide promising support for its inclusion in ERAS protocols, although further high-quality trials are needed.

**Registration:**

PROSPERO ID: CRD42024585785.

## Introduction

The perioperative management of pediatric cardiac surgery is critical for modulating the physiological response to surgical stress and influencing postoperative outcomes. Triggers such as median sternotomy, tissue trauma, and surgical drains activate the sympathetic and endocrine systems, resulting in a heightened stress response.^1^ Inadequate analgesia may lead to complications like delayed recovery, respiratory compromise, psychological distress, and increased risk of chronic pain syndromes.^2^, ^3^, ^4^

Opioid-based regimens have traditionally been the cornerstone of pain management but often provide suboptimal analgesia and cause side effects such as delayed extubation, respiratory and cardiovascular issues, nausea, pruritus, and opioid dependence.^5^^,^^6^ Despite advances, significant variability remains in managing postoperative pain in these patients.^7^ Multimodal analgesia, central to Enhanced Recovery After Surgery (ERAS) protocols, aims to reduce opioid reliance while optimizing pain control.^4^ Neuraxial anesthesia, although effective, is limited by risks like hemodynamic instability and perimedullary hematoma, especially in patients undergoing cardiopulmonary bypass and those with postoperative coagulopathy.^8^ Consequently, regional nerve blocks have emerged as promising alternatives for pain control and opioid minimization, with improved recovery outcomes.^9^^,^^10^ Advances in perioperative ultrasound have further enhanced their safety and efficacy in pediatric settings.^6^

Most studies have focused on individual techniques rather than direct comparisons, limiting the understanding of nerve blocks’ roles within multimodal analgesia bundles and the development of evidence-based ERAS protocols.^10^ This study aims to systematically evaluate and compare the efficacy of preemptive regional nerve blocks in pediatric cardiac surgery using Bayesian network meta-analysis, focusing on pain control, opioid consumption, and recovery outcomes.

## Methods

This systematic review and Bayesian NMA was registered under the PROSPERO database (CRD42024585785) and adheres to the guidelines outlined in the Cochrane Handbook, following the criteria recommended by PRISMA-NMA guidelines.^11^, ^12^, ^13^

### Search methods

A comprehensive literature search was conducted across multiple electronic databases, including Medline, Embase, Web of Science, and Cochrane Library, from September 4 to October 2024, without language restriction. The search strategy was initially developed and rigorously tested in Medline, then adapted for the other databases maintaining core structure and logic (Supporting Information S3). Additionally, searches of relevant clinical trial registries identified ongoing trials and study protocols. We contacted authors to inquire about unpublished results or additional data. References from included studies were screened to capture potentially relevant articles. Finally, we removed the duplicates among the identified documents using Rayyan.^14^

### Eligibility criteria

#### Inclusion criteria

Eligible studies included Randomized Controlled Trials (RCTs) published in peer-reviewed journals that evaluated pediatric patients (0‒12 years) undergoing cardiac surgery by sternotomy and receiving preemptive regional nerve blocks. Studies were required to report pain, or opioid consumption, or recovery outcomes. Studies were required to be prospectively registered in a national or international clinical trials database. There were no restrictions on the publication date for inclusion or language of publication. The eligibility criteria were designed to maximize transitivity by ensuring that included populations, interventions, and outcomes were sufficiently comparable across studies.

#### Exclusion criteria

We excluded non-randomized studies, studies without protocols, and studies that performed postoperative regional blocks. We also excluded studies involving patients undergoing cardiac procedures without comparable pain stimulus, such as percutaneous interventions, pacemaker implantation, or catheter-based procedures.

### Interventions

#### Intervention group

The interventions considered included the following regional nerve blocks: Erector Spinae Plane Block (ESPB), Medial Transversus Plane Block (MTPB), Multiple Injection Costotransverse Block (MICB), Pectoral Interfacial Block (PIFB), Thoracic Paravertebral Block (TPVB), Thoracic Retrolaminar Block (TRLB), and Transversus Thoracis Muscle Plane Block (TTPB).

#### Comparator group

Placebo and non-placebo control groups were initially analyzed as separate nodes. However, sensitivity analyses demonstrated that Surface Under the Cumulative Ranking (SUCRA) scores, effect estimates, and the overall ranking of interventions remained consistent when these groups were merged. Therefore, for the final model, they were combined into a single reference node ('NoBlock') to facilitate interpretation and streamline comparisons across treatment strategies.

### Outcomes evaluated

The primary outcomes of interest in this review included extubation time, intraoperative fentanyl-equivalent consumption, and pain scores at 12 hours postoperatively. Secondary outcomes included pain scores at 24 hours postoperatively, postoperative mean fentanyl-equivalent consumption at 24 hours, time to the first request for rescue analgesia, length of hospital and ICU stay, and the incidence of adverse effects, including Postoperative Nausea and Vomiting (PONV) and pruritus.

In the included studies, pain scores were reported using the Modified Observer's Pain Scale (MOPS), a 10-point scale with 1-point increments; the Face, Legs, Activity, Cry, Consolability Scale (FLACC), a 10-point scale with 2-point increments; the Visual Analogue Scale (VAS); and the Numeric Rating Scale (NRS).^15^

### Study selection and data extraction

Two authors (BW and GW) independently screened the titles and abstracts of all identified records based on the eligibility criteria. Full texts of potentially relevant citations were retrieved and evaluated for inclusion. Any discrepancies were resolved through consensus. Two reviewers (BW and GW) independently extracted data using a standardized spreadsheet in Google Sheets. When reported data was unavailable for direct extraction, the corresponding author was contacted for clarification. The primary data source consisted of numerical values presented in tables and figures. Data presented only in graphical format were extracted using WebPlot Digitizer version 4.7.^16^

### Statistical analysis

#### All analyses were conducted using R software, employing a Bayesian framework

Relative treatment effects were estimated for each outcome. Binary outcomes were reported as Risk Ratios (RR), while continuous outcomes were expressed as Mean Differences (MD). Standardized Mean Differences (SMDs) were used for pain scores to account for variation in measurement scales, while Mean Differences (MDs) were calculated for fentanyl-equivalent consumption.

#### Network modelling and consistency

Non-informative priors were used to minimize bias, ensuring that posterior estimates were primarily driven by observed data. Various configurations of iteration counts, burn-in periods, and thinning intervals were systematically tested to optimize precision and ensure model convergence. The most suitable configuration was selected based on convergence diagnostics, including Gelman-Rubin-Brooks diagnostics, trace plots, and auto correlograms, which confirmed adequate mixing, stable oscillations around the posterior mean, and low autocorrelation. Model adequacy was evaluated through posterior predictive checks and Deviance Information Criterion (DIC), with lower DIC values indicating superior model fit.

Final simulations were conducted using Markov Chain Monte Carlo (MCMC) techniques with a sufficiently high number of iterations. Node-splitting models were employed to assess incoherence between direct and indirect evidence.

To enhance graphical representations and to improve assessment of evidence confidence, we repeated all statistical analysis on the Confidence in Network Meta-Analysis (CINeMA) web application.^17^ Due to expected mean age differences between studies, we performed a covariate analysis with shared coefficients using the MetaInsight web application controlling for age for all included outcomes.^18^ The MetaInsight web application was also used to enhance the graphical representation of SUCRA scores by generating Litmus rank-o-grams, which integrate SUCRA values into rank distributions to visually summarize the relative performance of each treatment.^19^^,^^20^

The methodology presented here is not exhaustive. A detailed description of statistical analysis is available in Supporting Information S1, which provides detailed instructions for interpreting our methods and findings.

### Assessment of quality of evidence

#### Risk of bias

Two independent reviewers (BW and JA) assessed the methodological quality of the included trials using the Revised Cochrane Risk-of-Bias Tool for Randomized Trials (RoB 2).^21^ Any discrepancies were resolved by consensus, and if consensus could not be reached, a third reviewer (GW) was consulted.

#### Confidence in estimates

Confidence was evaluated using the CINeMA framework,^17^ which considers within-study bias (from the Risk of Bias assessment), reporting bias (including selective outcome reporting via Egger’s test and the Risk of Bias in Multi-Endpoint Network [RoB-MEN] framework), indirectness, imprecision, heterogeneity, and incoherence. Imprecision was determined by whether confidence intervals, derived from CINeMA framework, crossed the null effect or indicated opposing clinical effects.^22^ Heterogeneity was assessed using prediction intervals provided by CINeMA. Predefined thresholds for minimal clinically important differences were set as follows: an SMD of 1 for pain severity,^15^^,^^23^ an MD of 4 µg.kg^-1^ for fentanyl consumption,^24^ and a relative risk of 1.2 for increased adverse effects. Clinically relevant differences for key secondary outcomes were defined as a 4-hour difference for extubation time and time to rescue analgesia, a 6-hour difference for ICU stay, and 0.5 days for hospital stay. These thresholds provided a structured basis for interpreting the clinical significance of the observed effects.

#### GRADE assessment

The GRADE approach was employed to systematically evaluate the quality of evidence for each outcome, categorizing it into four levels: high, moderate, low, and very low.^25^ This assessment incorporated insights from CINeMA, integrating considerations of risk of bias, inconsistency, indirectness, imprecision, and publication bias. By combining these elements within the structured framework of GRADE, we provided a structured and transparent evaluation of the certainty in the estimated treatment effects.^17^^,^^21^^,^^25^

## Results

### Study selection

**Figure 1:**
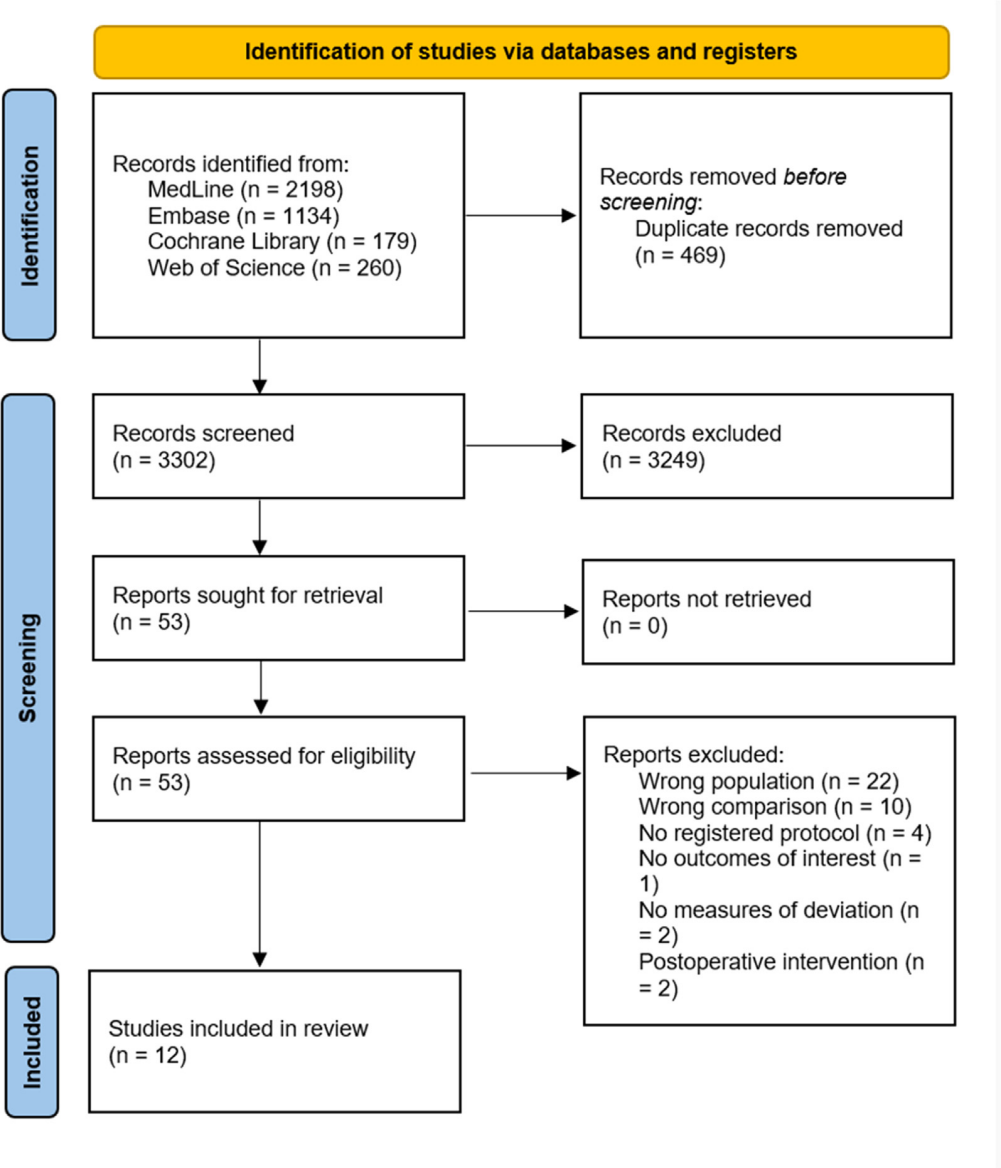
PRISMA flowchart.

Our comprehensive literature search identified 3,771 records. After the exclusion of 469 duplicates, 3,302 unique records remained. Title and abstract screening yielded 52 records for full-text review. Ultimately, 12 studies comparing seven different regional blocks in pediatric cardiac surgery, comprising 969 participants, met the inclusion criteria.^26^, ^27^, ^28^, ^29^, ^30^, ^31^, ^32^, ^33^, ^34^, ^35^, ^36^, ^37^ These studies included 11 two-arms studies,^26^^,^^27^^,^^29^, ^30^, ^31^, ^32^, ^33^, ^34^, ^35^, ^36^, ^37^ with 2 head-to-head comparisons,^26^^,^^27^ and one three-arm study,^28^ adding one more head-to-head comparison. No study was excluded solely due to lack of prospective registration, as all studies meeting the remaining inclusion criteria were registered and therefore eligible for inclusion (Fig. 1).

### Study characteristics

The characteristics of the included studies are detailed in Supporting Information S3. The mean (Standard Deviation [SD]) sample size across the studies was 80.8 (37.2), with a mean (SD) age of 3.71 (2.88) years. Of the total participants, 50.2% were female. The mean (SD) length of surgery was 149.30 (46.5) minutes. All twelve trials were conducted between 2020 and 2024, predominantly in Egypt (58.3%), followed by China (16.7%), India (16.7%), and Turkey (8.3%).

### Risk of Bias

#### Synthesis of results

**Figure 2:**
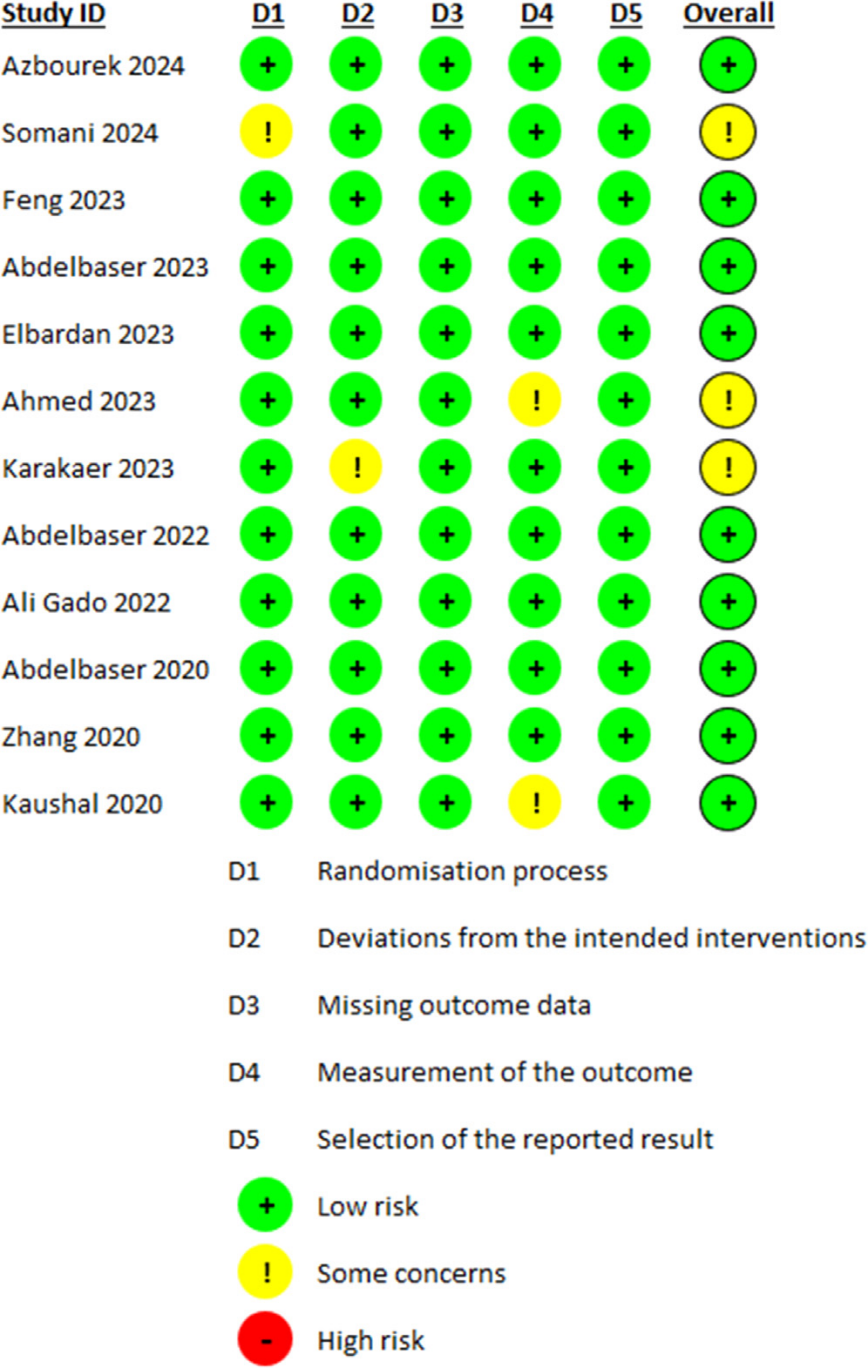
Risk of bias plot.

Opioid consumption Fig. 2

Post-operative cumulative fentanyl-equivalents consumption at 24 hours

Analysis included seven studies, involving 434 patients. Five studies reported fentanyl consumption, and two studies reported morphine consumption. TRLB ranked highest for reducing fentanyl-equivalent consumption (SUCRA 82.6%, MD -9.66 μg.kg^-1^, 95% CrI -21.12 to 1.69). TTPB ranked second (SUCRA 78.7%, MD -8.61 μg.kg^-1^, 95% CrI -20.04 to 2.83) ‒ Table 1.

**Table 1:** Bayesian network summary of findings: fentanyl-equivalent consumption.

Time to rescue analgesia

Analysis included seven studies, involving 563 patients. TRLB ranked highest for delaying rescue analgesia (SUCRA 78%, MD 4.99 hours, 95% CrI -0.82 to 10.83). TTPB ranked second (SUCRA 65%, MD 3.99 hours, 95% CrI -1.85 to 9.79) ‒ Table 1.

Intraoperative fentanyl-equivalents consumption

Analysis included twelve studies, involving 969 patients. PIFB ranked highest for reducing intraoperative fentanyl consumption (SUCRA 91.4%, MD -40.91 μg.kg^-1^, 95% CrI -86.19 to 1.07). TTPB ranked second (SUCRA 68.2%, MD -19.37 μg.kg^-1^, 95% CrI -54.93 to 15.01). Supporting Information S3.

### Pain-scales

Pain scores were measured at rest or without specification, by FLACC scale (1 study),^29^ or MOPS (10 studies).^26^, ^27^, ^28^^,^^30^, ^31^, ^32^, ^33^^,^^35^, ^36^, ^37^ One study reported pain scores measured by VAS but did not provide deviation measurements.^34^

### Pain scores

At 12 hours

Analysis included ten studies, involving 720 patients. TTPB ranked highest for pain reduction (SUCRA 99.9%, SMD -4.39, 95% CrI -5.57 to -3.16), representing a clinically significant reduction in pain scores. ESPB, MICB and “No Block” had the lowest probability of reducing pain ‒ Table 1.

At 24 hours

Analysis included eight studies, involving 596 patients. TTPB ranked highest for pain reduction at 24 hours (SUCRA 91.9%, SMD -1.97, 95% CrI -3.28 to -0.65). PIFB ranked second (SUCRA 78.2%, SMD -1.58, 95% CrI -2.89 to -0.28) — Table 2.

**Table 2:** Bayesian network summary of findings: pain management.

### Recovery outcomes

#### Extubation time

Analysis included nine studies, involving 707 patients. TRLB ranked highest for reducing extubation time (SUCRA 88.4%, MD -3.47 hours, 95% CrI -7.85 to 0.91). TTPB ranked second (SUCRA 78.6%, MD -2.25 hours, 95% CrI -5.36 to 0.8). ESPB and MTPB had moderate SUCRA scores. Lower-ranked interventions like ESPB and PIFB had the lowest probability of reducing extubation time (Table 3).

**Table 3:** Bayesian network summary of findings: postoperative recovery ‒ extubation time and time of ICU Stay.

#### ICU stay

Analysis included twelve studies, involving 791 patients. TTPB ranked highest for reducing ICU stay (SUCRA 86.9%, MD -6.93 hours, 95% CrI -11.57 to -2.35). TRLB was also likely to contribute to a reduction in ICU stay (SUCRA 80.3%, MD -6.5 hours, 95% CrI -13.11 to 0.15). Lower-ranked interventions, such as PIFB and No Block, were likely to have minimal impact on reducing ICU stay length (Table 3).

#### Hospital stay

The analysis included four studies, involving 410 patients. TTPB ranked highest for reducing hospital stay (SUCRA 95.5%, MD -2.5 days, 95% CrI -5.12 to 0.11). TPVB was likely to have a minimal impact on reducing Hospital Stay. Supporting Information S3.

### Adverse effects

#### PONV Incidence

PONV was reported in nine studies, involving 779 patients. ESPB ranked highest for reducing PONV risk (SUCRA 76.9%, RR 0.41, 95% CrI 0.11 to 1.35). PIFB ranked lowest. Supporting Information S3.

#### Pruritus incidence

Pruritus was reported as an outcome in six studies, involving 394 patients. TPVB ranked highest for reducing pruritus (SUCRA 68.1%, RR 0.45, 95% CrI 0.05 to 3.96). ESPB ranked second (SUCRA 56.9%, RR 0.63, 95% CrI 0.08 to 4.29). TTPB ranked lowest. Supporting Information S3.

#### Other adverse events

Other adverse events were sparse and inconsistently documented. Isolated cases of pneumothorax, paravertebral hematoma, fever, bradycardia, hypotension, respiratory depression, reintubation, local anesthetic toxicity, and neurological deficits were assessed. Supporting Information S3 provides a detailed description of these adverse events, including absolute frequencies by intervention group.

### Cumulative SUCRA scores

**Figure 3:**
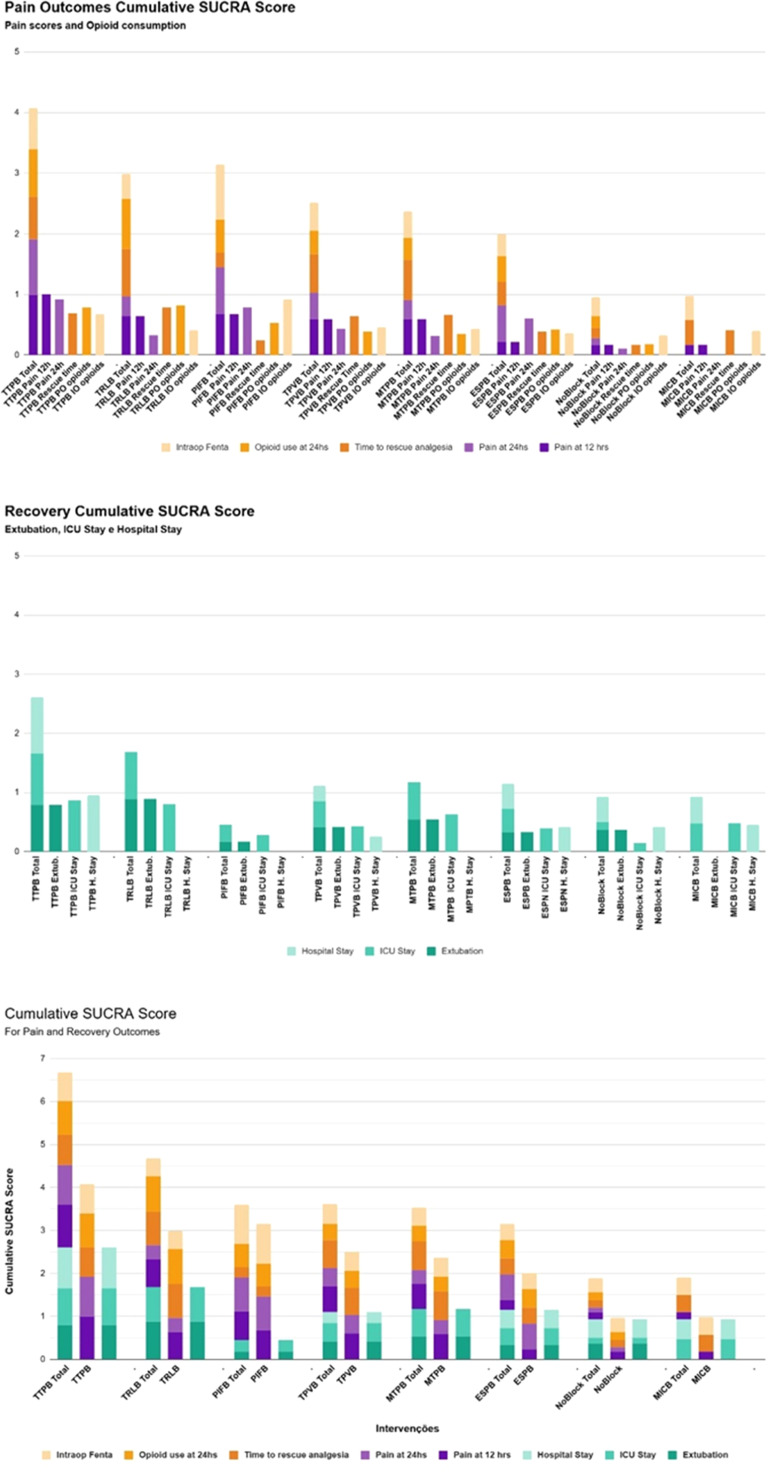
Cumulative SUCRA scores for pain outcomes highlight TTPB as the most effective intervention across all metrics, including pain at 12/24 hours and opioid consumption (intraoperative and at 24 hours). TRLB and PIFB demonstrate moderate efficacy, while lower-ranked blocks, such as MICB and NoBlock, show minimal impact on pain and opioid-related outcomes. Cumulative SUCRA scores for recovery outcomes (extubation, ICU stay, hospital stay) show TTPB as the top-ranking intervention, followed by moderate performance from TRLB. Lower-ranked blocks, such as MICB and NoBlock, exhibit minimal contributions to recovery metrics. Cumulative SUCRA scores demonstrate TTPB's probabilistic better performance across different outcomes, including pain at 12/24 hours, hospital/ICU stay, and intraoperative fentanyl use. ESPB, Erector Spinae Plane Block; Fenta, Fentanyl; ICU, Intensive Care Unit; Intraop, Intraoperative; MTPB, Medial Transversus Plane Block; MICB, Multiple Injection Costotransverse Block; PIFB, Pectoral Interfacial Block; SUCRA, Surface Under the Cumulative Ranking; TPVB, Thoracic Paravertebral Block; TRLB, Thoracic Retrolaminar Block; TTPB, Transversus Thoracis Muscle Plane Block.

Figure 3 presents a comprehensive overview of comparative intervention performance by combining SUCRA data for pain management and recovery parameters.

Each bar’s height corresponds to the probability that a given treatment ranks among the most effective options for its respective outcome. Interventions exhibiting taller bars across multiple domains suggest a more consistent benefit probability. However, not all interventions contributed data for every outcome. As a result, some bars may appear shorter due to incomplete outcome reporting. This limitation underscores the need for cautious interpretation, as the comparative performance of certain interventions may be underestimated due to incomplete data availability across domains. Ranking interpretation should also consider the corresponding effect sizes and their uncertainty. Further details on SUCRA calculations and individual outcome rankings, including Litmus rank-o-grams, are available in Supplementary Information S2 and S4 (Fig. 3).

### Heterogeneity and transitivity evaluation

In node‑splitting analyses (Supporting Information S4), only three comparisons ‒ TTPB versus NoBlock, PIFB versus NoBlock and TTPB versus PIFB for intraoperative fentanyl consumption ‒ showed discrepancies between direct and indirect estimates, while overall outcome heterogeneity remained low (I² ranged from 0% to 11%). Despite these discrepancies, we found no discernible link between the inconsistent comparisons and any clinical or methodological characteristic.

As detailed in Table 1, key effect modifiers ‒ mean patient age (1.5–7 years), sternotomy technique, and block‑specific protocols (local anesthetic type, volume, ultrasound approach and timing) ‒ were evenly distributed across all studies, with no clear link to the inconsistent comparisons. Uniform preemptive block administration, blinded outcome assessment and standardized methodology further support the transitivity assumption.

### Covariate analysis

Although our protocol initially planned for covariate adjustments in the network meta-analysis, the limited number of studies relative to the number of interventions precluded their reliable inclusion. Conducting a meta regression in this context would have increased the risk of overfitting, yielded unstable estimates with wide credible intervals, and compromised the robustness of the findings, potentially leading to misleading conclusions. The meta-regression conducted using MetaInsight web application reflected these limitations, showing inconsistent trends and wide credible intervals.^18^ For instance, the direction of age-related effects differed at 12 and 24 hours, despite stable treatment rankings. This inconsistency suggested that the observed trends were not robust and could be misleading.

To avoid overinterpretation of inconclusive results, we decided not to present the age-adjusted results. Therefore, our primary conclusions were based solely on the main network meta-analysis without age adjustment.

This limitation, stemming from the paucity of available studies rather than a methodological choice, prevented an objective evaluation of age as an effect modifier. Nonetheless, mean patient age was relatively homogeneous across trials ‒ eight studies reported means between 4.29 and 7 years, and four studies between 1.30 and 2.49 years. However, given the broad age range of 0–12 years, the potential for residual confounding by age cannot be entirely excluded and should be considered when interpreting the results.

## Discussion

This network meta-analysis compared regional nerve blocks in pediatric cardiac surgery, highlighting variability in their performance across outcomes such as analgesia, opioid consumption, recovery, and adverse effects. TTPB consistently ranked among the most effective techniques for pain relief and recovery. Other blocks, such as TRLB and TPVB, also showed notable performances, particularly in pain relief and recovery metrics, suggesting their potential role in optimizing postoperative care in pediatric cardiac surgery.

Postoperative pain after cardiac surgery is multifactorial, with median sternotomy causing intense discomfort due to tissue disruption, rib retraction, and sternal manipulation. Inadequate pain control can impair respiratory function, increasing the risk of complications such as atelectasis, pneumonia, and prolonged mechanical ventilation ^4^ The anterior chest wall is mainly innervated by the anterior branches of intercostal nerves (T2‒T6), while irritation from surgical drains and rectus abdominis involvement further contribute to pain.^38^ Reducing opioid consumption, facilitating early extubation, and accelerating overall recovery rely on effectively targeting these neural pathways, particularly through regional nerve blocks.

Pediatric patients with congenital heart disease frequently require multiple surgical interventions, resulting in cumulative opioid exposure and a heightened risk of tolerance, dependence, and long-term adverse effects. Effective strategies that reduce opioid consumption while maintaining adequate analgesia are essential to optimize perioperative care, and regional anesthesia techniques have emerged as a key component of such multimodal approaches. TTPB, by targeting the anterior branches of the intercostal nerves, provides effective analgesia for the sternum and anterior chest wall, making it particularly beneficial in managing pain after median sternotomy. TRLB involves the injection of local anesthetic into the retrolaminar plane, adjacent to the dorsal surface of the thoracic vertebrae, allowing for spread to the paravertebral space and resulting in analgesia of the posterior and lateral thoracic wall.

Early extubation is a critical postoperative objective, associated with reduced cardiopulmonary complications, shorter Intensive Care Unit (ICU) stays, and improved hemodynamic stability.^39^ Spontaneous breathing diminishes the need for fluid resuscitation and inotropic support while mitigating the adverse effects of prolonged mechanical ventilation.^39^^,^^40^ These benefits are particularly relevant in cavopulmonary surgeries ‒ such as the Glenn or Fontan procedures ‒ where maintaining spontaneous breathing enhances cardiac output.^2^^,^^39^^,^^41^ However, achieving early extubation requires adequate analgesia to prevent agitation, which can increase the risk of bleeding and cardiovascular instability.^41^ Moreover, inadequate analgesia can delay extubation, prolong mechanical ventilation, and increase the likelihood of complications such as ventilator-associated pneumonia. Notably, extubation failure has been linked to a tenfold increase in postoperative mortality.^39^

Beyond immediate postoperative concerns, prolonged hospitalization and delayed recovery pose additional risks, particularly in neonates and infants, whose immature brains are more susceptible to neurotoxicity.^42^ Notably, over 40% of children with congenital heart defects exhibit preoperative brain injuries, and approximately one-third sustain new neurological postoperative injuries.^42^^,^^43^ Therefore, optimizing analgesia and expediting recovery are critical components in mitigating these neurodevelopmental risks.

This study also underscores the distinct profiles of individual regional nerve blocks. For example, the PIFB ranked well in terms of postoperative pain management in cardiac surgery. However, the recovery metrics in this analysis ‒ such as time to extubation, ICU stay, and hospital length of stay ‒ reflect broader aspects of recovery beyond pain control alone. Despite effective analgesia, PIFB showed a potentially limited impact on these recovery outcomes. By targeting the intercostal nerves through the injection of local anesthetic between the pectoralis major and intercostal muscles, PIFB provides analgesia to the anterior chest wall.^44^ However, its limited performance on recovery metrics suggests that PIFB may not fully address the complex factors influencing postoperative recovery following cardiac surgery.

Selecting specific interventions aims to optimize both pain relief and recovery outcomes. Techniques such as TTPB and TRLB demonstrated a favorable balance, effectively combining analgesia with improved recovery metrics. These findings highlight the importance of tailoring regional block strategies to individual patients by accounting for factors such as comorbidities, surgical approaches, and patient-specific needs. Such an individualized approach is essential for providing the best care for pediatric patients.

Although adverse effects were infrequently reported, their evaluation remains critical for assessing the safety of regional blocks.^45^ The variability in adverse event reporting across studies highlights the need for standardized safety assessments in future trials. While this NMA provides comparative insights into the performance of different blocks, further data on adverse events is necessary to provide a more precise understanding of the risk-benefit balance associated with each technique.

### Strengths of the study

This review has several strengths, including a pre-registered protocol, a comprehensive literature search that encompassed trial protocols, and rigorous methodology, with duplicate and independent screening and data extraction. The network meta-analysis integrated both direct and indirect evidence, while GRADE assessments evaluated the certainty of the findings. High transitivity was observed, with comparable mean ages across studies and all procedures involving sternotomies. Furthermore, low heterogeneity enhances the reliability of results. Collectively, these factors establish this study as the most comprehensive and up-to-date synthesis of evidence on regional blocks for sternotomy in pediatric cardiac surgery.

### Limitations

Despite these strengths, certain limitations should be acknowledged. Most included studies were conducted in a limited geographical scope, with a concentration in a few countries (notably Egypt, India, and China), which may affect the generalizability of the findings. In addition, small sample sizes were common, further limiting generalizability and also reducing the precision of effect estimates. Additionally, the absence of age-stratified analyses in most studies may limit applicability across pediatric subgroups.^46^, ^47^, ^48^ Long-term outcomes, particularly chronic postoperative pain, were insufficiently assessed, representing a gap in current evidence. Furthermore, adverse events were inconsistently reported across studies, often without clear definitions or standardized timeframes, which hinders a robust comparative safety assessment of the interventions analyzed.

Future research should address these limitations to enhance the reliability and applicability of findings. Large-scale, multicenter studies are needed to ensure adequate sample sizes, increase statistical power and reduce the risk of type II errors. Stratification by surgery type and pediatric age groups is essential to capture developmental differences in pain perception and recovery and to minimize age-related bias. Furthermore, standardized reporting of long-term outcomes, including chronic postoperative pain, should be incorporated into study protocols to provide a more comprehensive understanding of postoperative recovery. Addressing these gaps will strengthen the evidence base and advance pediatric cardiac surgery practices.

## Conclusion

This network meta-analysis identified several effective regional analgesia techniques for pediatric cardiac surgery. While credibility intervals overlapped in some comparisons, TTPB consistently ranked among the most effective across multiple outcomes. These findings align with ERAS principles, supporting improved pain management, reduced opioid consumption, and enhanced recovery. By optimizing regional block strategies, this evidence may inform the refinement of perioperative protocols and advance pediatric surgical care. Nonetheless, prospective, multicenter randomized controlled trials with age-stratified analyses are warranted ‒ particularly to assess long-term outcomes such as chronic postoperative pain and potential neurodevelopmental effects.

## Conflicts of interest

The authors declare no conflicts of interest.
